# Efficacy of magnetic resonance imaging with an SPGR sequence for the early evaluation of knee cartilage degeneration and the relationship between cartilage and other tissues

**DOI:** 10.1186/s13018-019-1172-3

**Published:** 2019-05-24

**Authors:** Xin Yang, Zhuoyang Li, Yongping Cao, Yufeng Xu, He Wang, Licheng Wen, Zhichao Meng, Heng Liu, Rui Wang, Xiang Li

**Affiliations:** 1 Department of Orthopedics, Peking University First Hospital, No. 8, Xishiku Street, Beijing, 100034 Beijing China; 2 Department of Orthopedics, Zhejiang University School of Medicine First Affiliated Hospital, No. 79, Qingchun Road, Hangzhou, 310000 Zhejiang China; 3 Department of Radiology, Peking University First Hospital, No. 8, Xishiku Street, Beijing, 100034 Beijing China

**Keywords:** Osteoarthritis, Magnetic resonance imaging, Spoiled gradient-recalled sequence, Cartilage lesions

## Abstract

**Rationale and objectives:**

The aim of this study was to investigate the efficacy of magnetic resonance imaging (MRI) with a spoiled gradient-recalled (SPGR) sequence to evaluate early knee cartilage degeneration and the relationship between cartilage and other tissues using a modified Whole-Organ Magnetic Resonance Imaging Score (WORMS).

**Materials and methods:**

Eighty-four patients with knee joint pain were evaluated by X-ray and MRI with an SPGR sequence from June 2015 to December 2016. Joint degeneration was graded by two experienced radiologists using the Kellgren-Lawrence (K-L) grading scale. The modified WORMS was used to evaluate cartilage lesions, bone marrow abnormalities, bone cysts, osteophytes, joint effusion and synovitis. The difference between the WORMS of the SPGR and the T2 sequences evaluated by the Wilcoxon signed-rank test was determined, and the relationships between the WORMS features were evaluated by a Spearman correlation.

**Results:**

The modified WORMS for the cartilage lesion evaluation was significantly higher with the SPGR sequence than with the T2 sequence (*P* < 0.05). The cartilage lesions showed a moderate correlation with osteophytes, synovitis and joint effusion (Rs > 0.40, *P* < 0.05) and weak correlations with bone marrow abnormalities and bone cysts (Rs < 0.4, *P* < 0.05).

**Conclusion:**

The modified WORMS evaluation using MRI with the SPGR sequence was much better than the normal sequence for early knee osteoarthritis (OA). The cartilage lesions are associated with bone marrow abnormalities and the other features of OA.

## Introduction

Magnetic resonance imaging (MRI) is a common noninvasive imaging diagnostic technique for soft tissue. However, cartilage lesions cannot be distinctly observed via the typically used MRI sequences [1]. The three-dimensional fat-suppressed spoiled gradient-recalled (3D-FS-SPGR) sequence, which has a high contrast/noise ratio (CNR), can improve the contrast between the cartilage and the surrounding bone marrow by fat suppression to better display the cartilage [2]. There are few studies that report the usage of the SPGR sequence for the Whole-Organ Magnetic Resonance Imaging Score (WORMS). The purpose of this paper is to investigate the diagnostic accuracy of a modified WORMS using MRI with the SPGR sequence for the evaluation of early knee osteoarthritis (OA).

## Materials and methods

### Subjects

MRI with the SPGR sequence and radiographs from 84 patients were collected between June 2015 and December 2016. This study was approved by the Ethics Committee of our hospital. The REB number was (2014[713]). The patients who had chronic mild knee pain and met the American College of Rheumatology guidelines for knee OA [3] were included. The inclusion criteria were as follows: (1) patients had mild knee pain with Visual Analogue Scale (VAS) scores between 2 and 4 cm [4]; (2) at least 3 months of pain duration [5], and (3) the severity of radiographic OA was between grade 0 and 3 graded with the Kellgren-Lawrence (K-L) grading scale [6]. The exclusion criteria were as follows: (1) known diagnosis of inflammatory or metabolic diseases, such as rheumatoid arthritis, gout, tuberculosis or septic arthritis; (2) neoplastic diseases of the examined knee; (3) intra-articular bone fracture and ligament rupture of the knee joint; and (4) recent treatment prior to the imaging study. The mean age was 48.9 years (17–75 years). Thirty-four patients (40.48%) were men, and 50 patients (59.52%) were women. The left knee was involved in 37 patients (44.05%), and the right knee was involved in 47 patients (55.95%).

### MRI parameters

The patients were told to assume a supine position, and the knee joints were held in the centre of the coil to maintain their position during the examination. We used a 3.0 Tesla whole-body GE MR750 scanner (General Electric Healthcare, Milwaukee, WI) with an 8-channel knee coil. The imaging sequences included Cor-PDw (SE 2500/35 [TR msec/TE msec], 18-cm field of view [FOV], 4 mm/1 mm [slice thickness/interslice gap], 320 × 256 matrix, echo train length [ETL] of 10), Sag-T1w (655/12, 18-cm FOV, 4 mm/0.4 mm, 384 × 256, 3), Ax-T2w (2500/42, 18-cm FOV, 4 mm/1 mm, 384 × 256, 12), sagittal PD/T2w dual echo (2500/30/85, 18-cm FOV, 4 mm/0.4 mm, 352 × 256, 12) and 3D-FS-SPGR (7.5/2.9, 18-cm FOV, 2 mm/0 mm, 256 × 256 matrix, 15° flip angle).

### Data analysis

After two hours of training for performing the WORMS, two experienced registered radiologists who had 10 years of work experience independently evaluated the radiographs and MRI data.

### Radiographs

The severity of radiographic OA was graded via the K-L grading scale as follows: 0 = normal; 1 = possible joint space narrowing and osteophyte formation; 2 = definite osteophyte formation with possible joint space narrowing; 3 = multiple osteophytes, definite joint space narrowing, sclerosis and possible bony deformity; and 4 = large osteophytes, marked joint space narrowing, severe sclerosis and definite bony deformity [6]. Each radiologist read the radiographs randomly to confirm the K-L grade. If there was a different decision from the two radiologists, the K-L grade was confirmed by our orthopaedic professor who had 30 years of work experience.

### MRI evaluation

The joint degeneration in knee OA was graded with the WORMS [7]. The knee joint was divided into four major regions: medial and lateral tibiofemoral joints, the patellofemoral joint and the portion of the tibia beneath the tibial spines (subspinous). Six features were evaluated using the WORMS, including the cartilage lesion, bone marrow abnormalities, bone cysts, osteophytes, synovitis and joint effusion. Cartilage lesions were graded in the four regions using the Recht scale [8]: 0 = normal, 1 = areas of inhomogeneous signal intensity of the articular cartilage on the sequences, 2 = defects involving less than half of the articular cartilage thickness, 3 = defects involving more than half of the cartilage but less than the full thickness and 4 = full-thickness defects exposing the bone. Synovitis was graded using the estimated maximal distention of the synovial cavity: 0 = normal, 1 = < 33% of maximum potential distention, 2 = 33–66% of maximum potential distention and 3 = > 66% of maximum potential distention [7].

Bone marrow abnormalities and bone cysts were graded on a 4-point scale: 0 = normal, 1 = < 25% of the region, 2 = 25 to 50% of the region and 3 = > 50% of the region [7]. Osteophytes and joint effusion were graded as follows: 0 = none, 1 = mild, 2 = moderate and 3 = severe [7].

### Statistical analysis

Statistical analysis was performed using the SPSS 23.0 software package (Chicago, IL, USA). Fleiss *κ* was used to compare inter-rater agreement for determining the K-L grade of the radiographic OA. The 95% confidence intervals for the *κ* values were calculated for the inter-observer reliability. The frequencies of the modified WORMS features, such as cartilage lesions, bone marrow abnormalities and bone cysts, were calculated when a patient had a score over 0 for different joint regions. The cartilage lesion scores and the WORMS were evaluated by the SPGR sequence and T2 sequence in different K-L grades and were analysed by the Wilcoxon signed-rank test. The difference was considered significant at *P* < 0.05. Spearman correlations were used to quantify the associations between the K-L grade and the sum of the WORMS in the primary analysis.

For the secondary analysis, the associations between cartilage lesions and other WORMS abnormalities were examined, and the sum of the cartilage lesion scores and other features were calculated for each region and compared amongst regions.

Spearman’s rho (Rs) was used to describe the Spearman correlations. We defined a 3-point scale as follows: 0–0.4 = weak correlation, 0.4–0.7 = moderate correlation and > 0.7 = significant correlation [9].

## Results

### X-ray and MRI findings

**Fig. 1:**
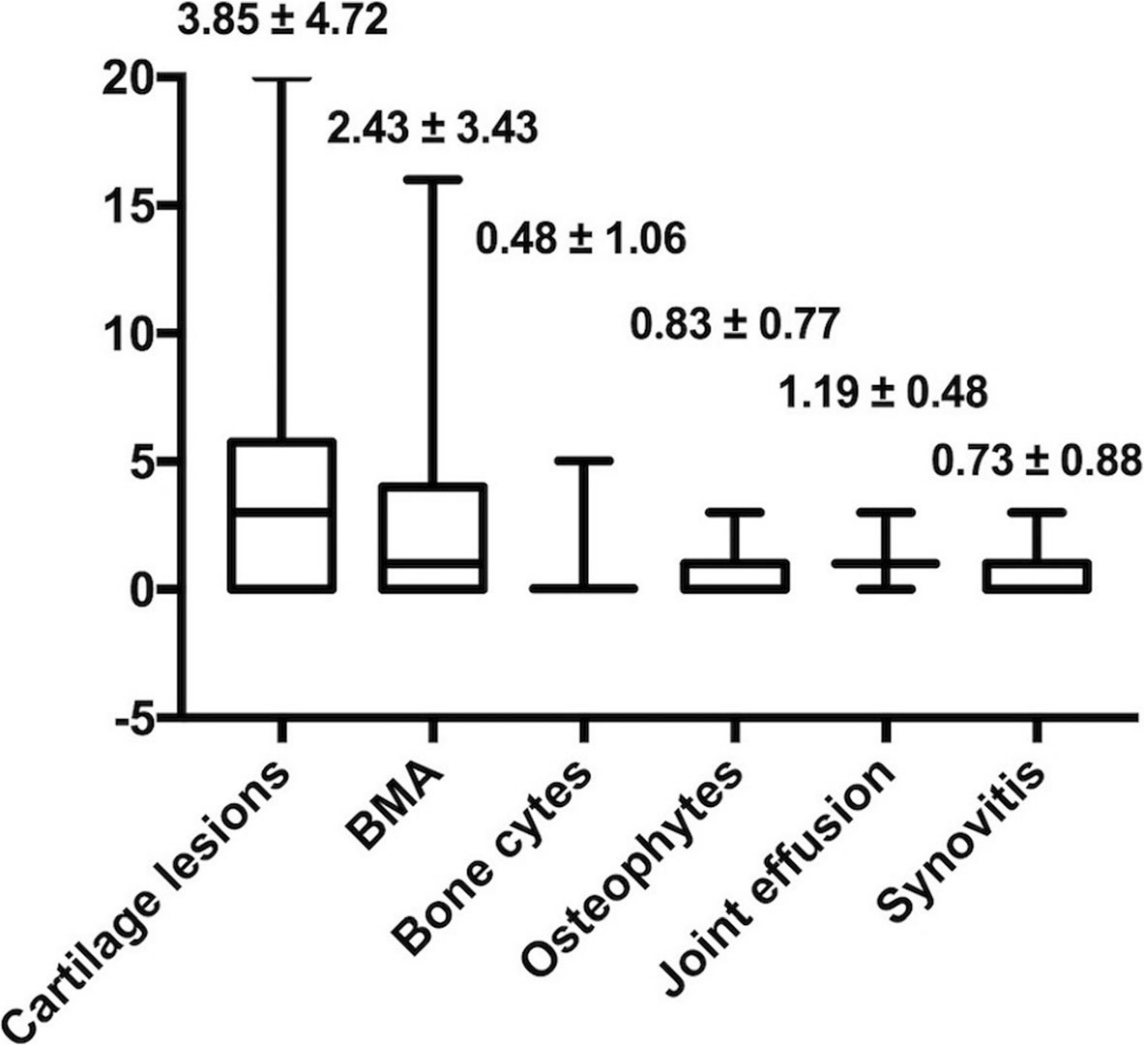
The mean WORMS. The mean WORMS values for the patients. BMA bone marrow abnormalities

The distribution of the K-L grading was as follows: grade 1, 36 cases; grade 2, 29 cases; and grade 3, 19 cases (Table 1). The Fleiss *κ* value was 0.77 (95% confidence interval, 0.73 to 0.82), indicating substantial agreement. According to the results of the MRI with the SPGR sequence, cartilage lesions, bone marrow abnormalities and bone cysts were approximately equally distributed amongst the different regions of the joint (Table 2). The highest frequency of cartilage lesions was in the patellofemoral joint (41 knees, 48.81%), while the lowest frequency was in the lateral tibiofemoral joint (15 knees, 17.86%). The subchondral bone marrow abnormalities were mostly present in the patellofemoral joint (33 knees, 39.29%) and rarely in the subspinous (8 knees, 0.95%). The results for the bone cysts showed that the patellofemoral joint had the highest frequency (8 knees, 9.52%), whereas both the lateral tibiofemoral joint and the subspinous had the lowest frequency (5 knees, 5.95%). The mean scores for the WORMS in the SPGR sequence are shown in Fig. 1. The mean score for cartilage lesions, subchondral bone marrow abnormalities, bone cysts, osteophytes, joint effusion and synovitis were 3.85 ± 4.72, 2.43 ± 3.43, 0.48 ± 1.06, 0.83 ± 0.77, 1.19 ± 0.48 and 0.73 ± 0.88, respectively.

**Table 1:** The K-L grading scale distribution for the patients

K-L grading	0	1	2	3
Total	0	36	29	19

*K-L* Kellgren-Lawrence

**Table 2:** The frequencies of the modified WORMS features in the different joint regions as determined with the SPGR sequence

WORMS feature	Regions			
	MFTJ	LFTJ	PFJ	S
Cartilage lesions	29 (34.52%)	15 (17.86%)	41 (48.81%)	
BMA	26 (30.95%)	19 (22.62%)	33 (39.29%)	8 (0.95%)
Bone cysts	7 (8.33%)	5 (5.95%)	8 (9.52%)	5 (5.95%)

*WORMS* Whole-Organ Magnetic Resonance Imaging Score, *SPGR* spoiled gradient-recalled, *MFTJ* Medial tibiofemoral joint, *LFTJ* lateral tibiofemoral joint, *PFJ* patellofemoral joint, *S* subspinous, *BMA* bone marrow abnormalities

### Comparison of cartilage scores for SPGR and T2 sequences

**Fig. 2:**
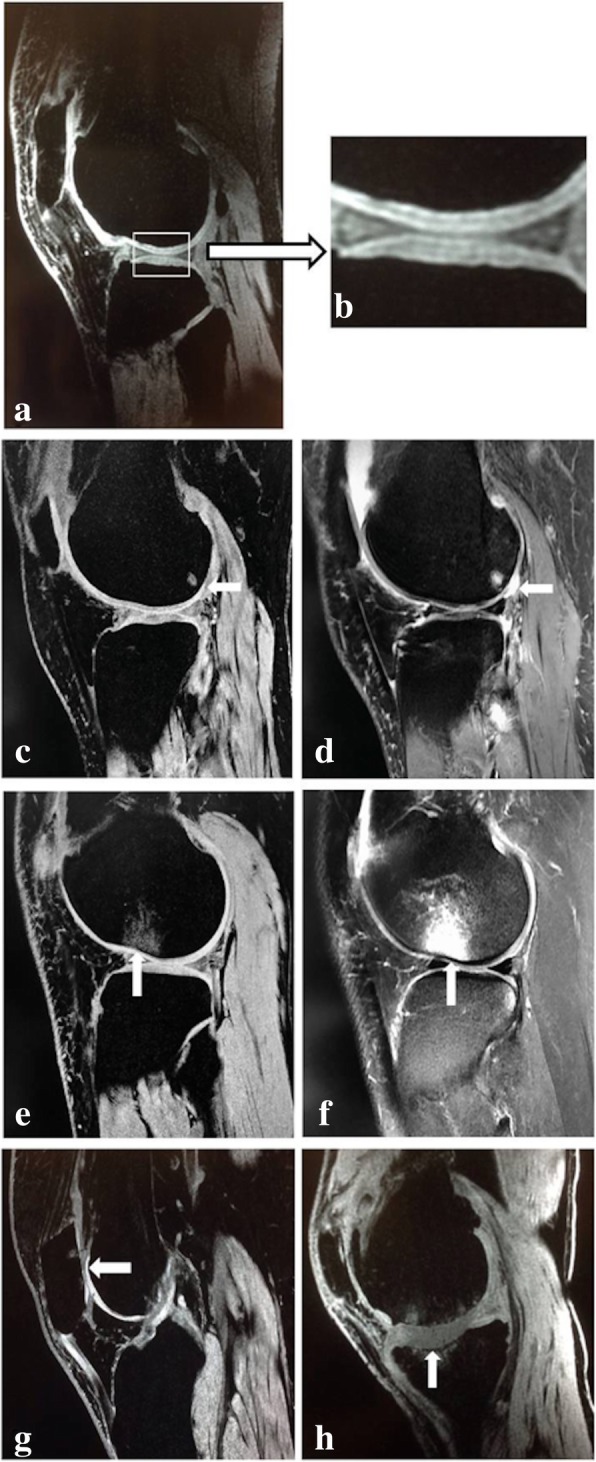
Normal cartilage and different grade lesions for the SPGR and T2 sequences. **a** and **b** show the five-layered cartilage structure of the tibial plateau and the three-layered cartilage structure of the femoral condyle. For the grade 1 cartilage lesion (arrow) in **c**, a low signal intensity for the cartilage distinctly appeared. The cartilage lesion (arrow) in **d** was covered by joint effusion, and the border between the cartilage and the joint effusion could not be distinguished with the T2 sequence. The articular cartilage layer disappeared, and the defect was less than 50% of the thickness of normal cartilage for a grade 2 cartilage lesion (arrow in **e**). The T2 sequence shown in **f** also shows a mixed signal for a cartilage lesion (arrow) and joint effusion. **g** shows a grade 3 patellar cartilage lesion (arrow) with a defect of up to 50% of the thickness of normal cartilage. **h** shows severe wear of the cartilage of the femur, tibia and patella, which is considered to be a grade 4 cartilage lesion, and the subchondral bone marrow exhibits a cystic or patchy high signal

According to the findings for the MRI with the SPGR sequence, five layers with different signal intensities were visible on the normal cartilage of the patella or femoral condyle (Fig. 2a, b). For grade 1 cartilage lesions, the low signal intensity in the cartilage and the high signal intensity in the subchondral bone appeared distinctly in the SPGR sequence (Fig. 2c). These represented a cartilage lesion and bone marrow abnormalities. However, in the T2 sequence, the cartilage lesion was covered by joint effusion, and the border between the cartilage and the joint effusion could not be distinguished (Fig. 2d). For the grade 2 lesions, the articular cartilage layer disappeared, the shape was irregular, and the defect was less than 50% of the normal cartilage thickness in the SPGR sequence (Fig. 2e). The T2 sequence also showed a mixed signal for cartilage lesions and joint effusion (Fig. 2f). Figure 2g shows the complete cartilage contours of the lateral femoral condyle, no abnormal signals in the cartilage, and a clear three-layer structure. However, the patellar cartilage showed grade 3 lesions, and the defect was up to 50% of the normal cartilage thickness in the SPGR sequence. A grade 4 cartilage lesion is shown in Fig. 2h. The cartilage of the femur, tibia, and patella was worn, and the subchondral bone marrow showed a cystic or patchy high signal.

The frequency and mean score for the cartilage lesions evaluated by MRI with the SPGR and T2 sequences are shown in Table 3. As the K-L grade of the X-rays increased, the frequency of cartilage lesions according to both SPGR and T2 sequences increased. In the K-L1 grade, the frequency evaluated by MRI with the SPGR sequence was 38.89%, and it was 19.44% when evaluated by MRI with the T2 sequence. The mean score for the cartilage lesion evaluation with the SPGR sequence was 1.19 ± 1.92, which was higher than that with the T2 sequence (0.39 ± 0.96) (*P* < 0.05) (Table 3). For the K-L2 grade, the frequencies of cartilage lesions were 72.41% and 58.62% using MRI with the SPGR and T2 sequences, respectively. The mean scores obtained with the SPGR and T2 sequences were 3.21 ± 2.79 and 1.76 ± 2.50, respectively; this difference was significant (*P* < 0.05) (Table 3). The frequency of cartilage lesions was the same when using the SPGR and T2 sequences in the K-L3 grade (89.47%); however, the score with the SPGR sequence was higher than that with the T2 sequence (9.84 ± 5.55 vs. 6.84 ± 5.04) (*P* < 0.05) (Table 3). The WORMS with the SPGR sequence for the K-L1, K-L2 and K-L3 grades were 4.78 ± 4.31, 8.38 ± 4.95 and 20.16 ± 7.90, respectively; with the T2 sequence, whereas with the WORMS, they were 4.06 ± 3.79, 7.00 ± 5.00 and 17.16 ± 7.59, respectively; and the differences in all grades were significant (*P* < 0.05) (Table 3).

**Table 3:** Comparison of frequencies of cartilage lesions, the cartilage lesion scores and the modified WORMS for the SPGR and T2 sequences for the different K-L grades

K-L grading scale			MRI sequence	
			SPGR	T2
K-L1 grade	Cartilage lesion	Frequency	14/36 (38.89%)	7/36 (19.44%)
		Mean ± SD	1.19 ± 1.92*	0.39 ± 0.96*
	WORMS		4.78 ± 4.31*	4.06 ± 3.79*
K-L2 grade	Cartilage lesion	Frequency	21/29 (72.41%)	17/29 (58.62%)
		Mean ± SD	3.21 ± 2.79*	1.76 ± 2.50*
	WORMS		8.38 ± 4.95*	7.00 ± 5.00*
K-L3 grade	Cartilage lesion	Frequency	17/19 (89.47%)	17/19 (89.47%)
		Mean ± SD	9.84 ± 5.55*	6.84 ± 5.04*
	WORMS		20.16 ± 7.90*	17.16 ± 7.59*
Total	Cartilage lesion	Frequency	52/84 (61.90%)	41/84 (48.80%)
		Mean ± SD	3.85 ± 4.72*	2.38 ± 3.86*
	WORMS		9.50 ± 8.11*	8.04 ± 7.30*

*P < 0.05. *WORMS* Whole-Organ Magnetic Resonance Imaging Score, *SPGR* spoiled gradient-recalled, *K-L* Kellgren-Lawrence, *MRI* magnetic resonance imaging

The mean score for the cartilage lesion evaluation with the SPGR sequence was 3.85 ± 4.72, which was higher than that with the T2 sequence (2.38 ± 3.86); this difference was significant (*P* < 0.05) (Table 3). The WORMS with the SPGR sequence was 9.50 ± 8.11, which was also higher than that with the T2 sequence (8.04 ± 7.30) (*P* < 0.05) (Table 3).

### Association between the K-L grade and the WORMS

**Fig. 3:**
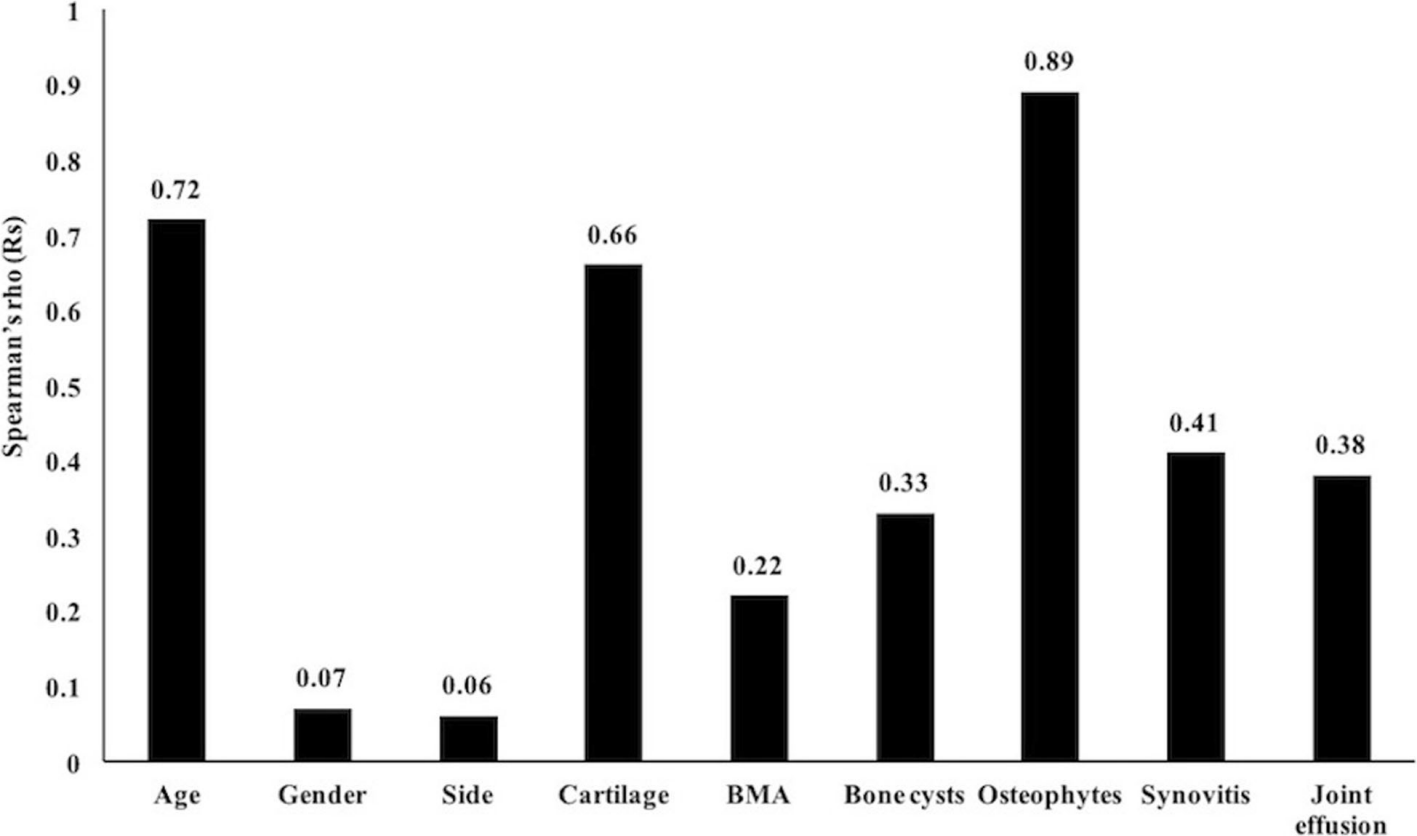
Correlation between K-L and WORMS. The cartilage lesions, osteophytes and synovitis showed moderate positive correlations with K-L, and weak positive correlation was observed between K-L and each of the bone marrow abnormalities, bone cysts and joint effusion. BMA bone marrow abnormalities

The results of the correlation analyses are shown in Fig. 3. The K-L grade was positively correlated with the patient’s age (Rs = 0.72, *P* < 0.01); however, no significant association between the K-L grade and gender or side was observed (*P* > 0.05).

The WORMS features were all positively correlated with the K-L grade. The osteophyte score significantly correlated with the K-L grade with an Rs of 0.89 (*P* < 0.05). The cartilage lesion score and the synovitis score with the SPGR sequence showed moderate correlation with the K-L grade with Rs values of 0.66 and 0.41, respectively (*P* < 0.05). A weak correlation was found between the K-L grade and bone marrow abnormalities, bone cysts and joint effusion scores with Rs values of 0.22, 0.33 and 0.38, respectively (*P* < 0.05).

### Association of cartilage lesions with other WORMS features

**Fig. 4:**
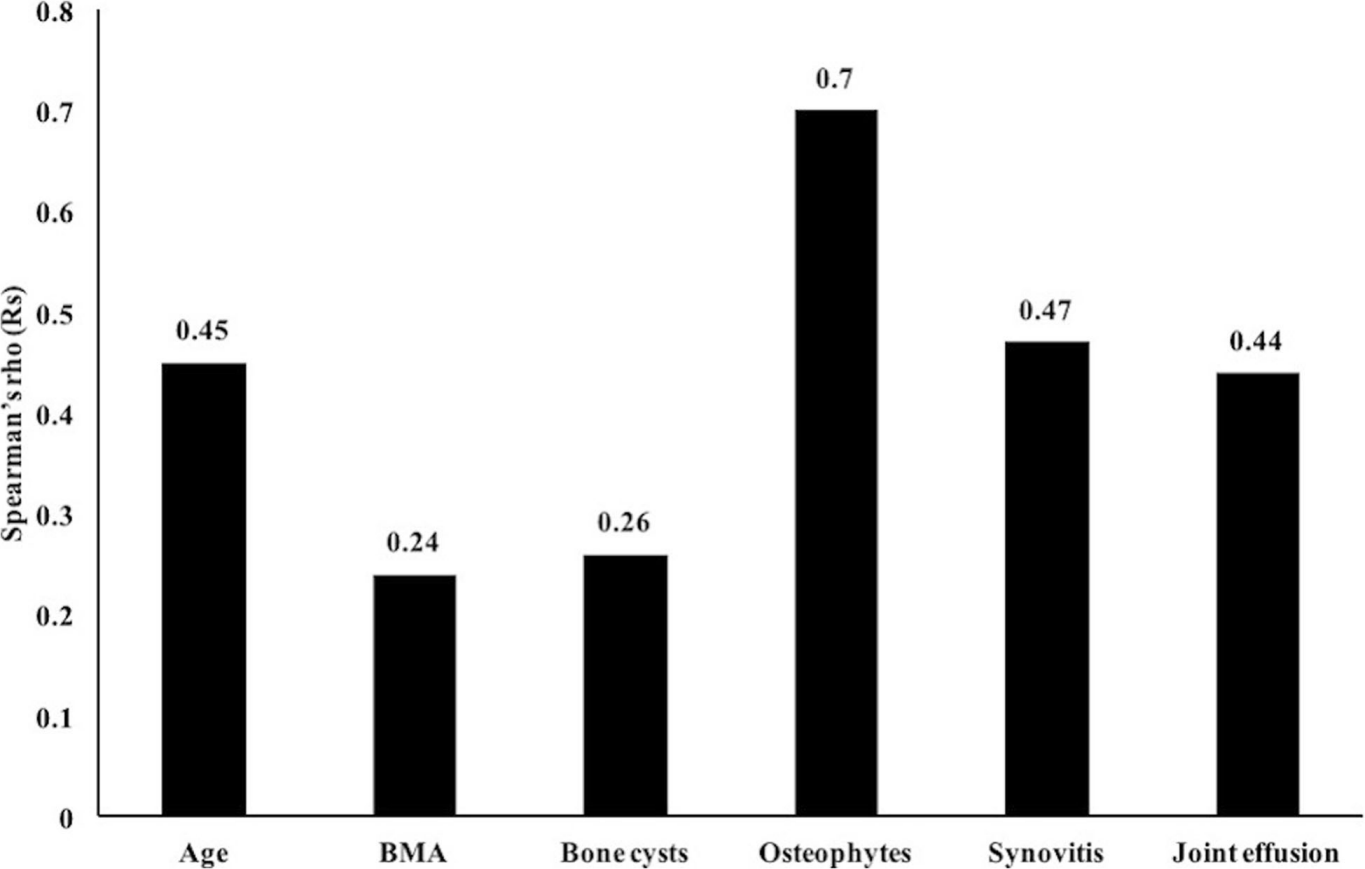
Correlation between cartilage lesions and other features. Moderate positive correlations were found between the cartilage lesions and each of the following: joint effusion, synovitis, osteophytes, bone cysts, bone marrow abnormalities and age. BMA bone marrow abnormalities

The severity of structural joint degeneration was expressed as the sum of WORMS with the SPGR sequence and ranged from 0 to 32 (mean of 9.50 ± 8.11). The cartilage lesion scores with the SPGR sequence ranged from 0 to 20 (mean of 3.85 ± 4.72). A weak correlation (Rs = 0.24, *P* < 0.05) was found between cartilage lesions and bone marrow abnormalities (Fig. 4). In 52 cases of articular cartilage lesions, the prevalence of subchondral bone marrow abnormalities was 71.15%. Of the 32 cases with normal articular cartilage, subchondral bone marrow abnormalities occurred in 40.63% of patients. A stronger moderate positive correlation was found between osteophytes and cartilage lesion scores (Rs = 0.70, *P* < 0.05). The analysis also showed that the cartilage lesion score (Rs = 0.47, *P* < 0.05) and the joint effusion score (Rs = 0.44, *P* < 0.05) were moderately correlated with synovitis. The bone cyst score weakly correlated with the cartilage lesion score (Rs = 0.26, *P* < 0.05) (Fig. 4).

## Discussion

Currently, in the early evaluation of knee cartilage degeneration, conventional MRI sequences can display the structure of the cartilage and subchondral bone but cannot detect abnormalities in cartilage layers during the early stage of OA [1].

Some studies indicate that in early OA, the moisture and proteoglycan content are altered, and collagen fibres begin to slightly wear and crack on the cartilage surface [10, 11]. These alterations do not cause changes in the contour of the articular cartilage; therefore, conventional MRI sequences cannot detect this abnormality. Zhao et al. [12] examined articular cartilage using the 3D-FS-SPGR sequence and found sensitivity, specificity and Kappa values of 93.1%, 98.3% and 0.849, respectively.

The main histological characteristic of articular cartilage is its layered structure [13], which includes four layers: the most superficial layer, the transitional layer, the radiation layer and the deepest calcification layer [14]. One study has shown that articular cartilage can present a “high–low–high signal” three-layered structure in the MRI, which is matched by histological stratification [15]. In our study, the use of MRI with the SPGR sequence showed that the normal articular cartilage was represented as a smooth circular arc band with a high signal. The obvious “high–low–high signal” three-layered structure could be detected, and even the five-layered structure could be detected. We considered the three high-signal layers to be the most superficial layer, the transitional layer and the radiation layer, and the low signal layers to be the interfaces between the two layers because of changes in the water and collagen content and tissue anisotropy [15]. The calcification layer was calcified under the tidemark. A previous study reported that the disappearance of the layered collagen structure was the earliest visible morphological change that occurs with OA [16]. Thus, we focused on low K-L grade patients to evaluate the effect of the SPGR sequence on the WORMS. We found that the differences in the cartilage scores and WORMS between the SPGR and T2 sequences were significant. The frequency of cartilage lesions evaluated by MRI with the SPGR sequence for both K-L1 and K-L2 grades was higher than that evaluated by MRI with the T2 sequence, and the differences in the mean scores for the cartilage lesions between the methods were significant. Even for the K-L3 grade, although the frequency of cartilage lesions was the same between the two approaches, the scores were significantly different. The results of our study demonstrate that the SPGR sequence is more sensitive than the T2 sequence for WORMS evaluation of early cartilage lesions and is advantageous for early detection of OA.

In our study, we found that the WORMS values were generally low with a mean score for cartilage lesions of only 3.85 ± 4.72. We speculate that the low K-L grade is mainly due to the age distribution of the patients, many of whom were young, which resulted in few patients who had moderate or severe OA or degeneration of the knee joints. However, most of the OA patients were found to have an initial onset in the patellofemoral joint, according to the WORMS evaluation. Cartilage lesions, bone marrow abnormalities and cysts exhibited the highest frequency in the patellofemoral joint, whereas the lowest frequency was observed in the intercondylar region. These results are consistent with previous literature [17] and demonstrate the accuracy of the WORMS system. Positive correlations were observed between the K-L grade and the features of the modified WORMS, which showed that MRI with the SPGR sequence is more comprehensive and effective than X-rays and MRI with the typically used normal sequence for the early evaluation of OA.

Many studies have observed an association between articular cartilage and subchondral bone and have demonstrated that the mechanical characteristics of subchondral bone and articular cartilage are related to the progression of OA [18–21]. These findings are consistent with the results of our study. We found that in the 52 patients who had mild cartilage lesions, the frequency of bone marrow abnormalities reached 71.15%, which indicated that these articular cartilage lesions were associated with bone marrow abnormalities. The correlation analysis between the features of the modified WORMS showed a positive correlation between cartilage lesions and bone marrow abnormalities. Therefore, we suggest that if conventional MRI sequences show signal changes in the subchondral bone marrow, cartilage lesions should be evaluated with the SPGR sequence.

To confirm negative physical examination of meniscus tears, MRI had been proved to lead to a 2.08 and 2.26 higher rate than patient history and physical examination alone and lead to more than a 10-fold lower rate of unnecessary surgeries than any other strategy to confirm positive physical examination [22]. Compared to the arthroscopy, MRI was suggested for the diagnosis and therapy of meniscal lesions in the economic and damage consideration [23, 24]. Alizai et al. [25, 26] suggested to use MRI-based semiquantitative grading methods to describe cartilage assessment and provide an update on the risk factors for cartilage loss in knee osteoarthritis. So the modified WORMS evaluation using MRI with the SPGR sequence is suitable for the evaluation of early knee arthritis and will have a bright future with the development of MRI technology.

## Conclusions

We used MRI with the SPGR sequence to determine the modified WORMS, which was better than that found with the conventional sequence for early OA. The SPGR sequence likely increases the sensitivity of the WORMS. Therefore, the SPGR sequence could be used for the evaluation of early knee arthritis in the clinical setting.

## Abbreviations

3FS-SPGR: Three-dimensional fat-suppressed spoiled gradient-recalled
BMA: Bone marrow abnormalities
CNR: Contrast/noise ratio
K-L: Kellgren-Lawrence
LFTJ: Lateral tibiofemoral joint
MFTJ: Medial tibiofemoral joint
MRI: Magnetic resonance imaging
OA: Osteoarthritis
PFJ: Patellofemoral joint
Rs: Spearman’s rho
S: Subspinous
WORMS: Whole-Organ Magnetic Resonance Imaging Score
